# Maximizing the Estrogenic Potential of Soy Isoflavones through the Gut Microbiome: Implication for Cardiometabolic Health in Postmenopausal Women

**DOI:** 10.3390/nu14030553

**Published:** 2022-01-27

**Authors:** Lindsay M. Leonard, Mun Sun Choi, Tzu-Wen L. Cross

**Affiliations:** Department of Nutrition Science, Purdue University, West Lafayette, IN 47907, USA; leonar25@purdue.edu (L.M.L.); mchoi@purdue.edu (M.S.C.)

**Keywords:** phytoestrogens, daidzein, (*S*)-equol, estrogen, microbiota, menopause, obesity, type 2 diabetes (T2D), insulin resistance, cardiovascular diseases (CVD)

## Abstract

Soy isoflavones have been suggested as an alternative treatment for managing postmenopausal symptoms and promoting long-term health due to their structural similarity to mammalian estrogen and ability to bind to estrogen receptors. Among all soy isoflavones and their metabolites, (*S*)-equol is known for having the strongest estrogenic activity. Equol is a metabolite of the soy isoflavone daidzein produced through intestinal bacterial metabolism. However, more than half of the human population is not able to produce equol due to the lack of equol-producing bacteria in their gastrointestinal tract. The interpersonal variations in the gut microbiome complicate the interpretation of data collected from humans. Furthermore, because rodents are efficient equol-producers, translatability between rodent models and humans is challenging. Herein, we first summarized the current knowledge of the microbial conversion of daidzein to equol, its relation to health, and proposed the need for developing model systems by which equol production can be manipulated while controlling other known confounding factors. Determining the necessity of equol-producing capacity within a gut microbial community when consuming soy as a functional ingredient, and identifying strategies to maximize equol production by modulating the gut microbiome, may provide future therapeutic approaches to improve the health of postmenopausal women.

## 1. Introduction

Menopause is an inevitable age-related loss of ovarian hormone production characterized by falling concentrations of estrogen and progesterone [1]. Menopause occurs at a median age of 50 years old and is a transitional stage that occurs for several years. Postmenopausal women are at significantly greater risk of obesity, Type 2 diabetes (T2D), cardiovascular diseases (CVD), osteoporosis, and breast cancer, which is thought to be driven by estrogen deficiency [2,3,4,5,6]. Paradoxically, estrogen replacement therapy can lead to unexpected cardiac events, greater risks of breast cancer, and acute liver disease, especially in those with a history of these diseases [7,8,9]. Alternative therapeutic strategies, particularly consumption of soy and soy isoflavones, have gained significant public attention due to the fear of potentially serious adverse effects from estrogen replacement therapy [10,11]. Compared to Western populations, a lower prevalence of postmenopausal-related symptoms and diseases in Asian populations has been attributed to greater soy consumption [12,13].

Soy isoflavones are plant-derived phytoestrogens that have structural similarity to mammalian-synthesized estrogen with anti-obesogenic and anti-atherosclerotic properties [14]. These phytoestrogens can bind to estrogen receptors ER-α and ER-β to act as estrogen agonists or antagonists [14]. Unlike artificial endocrine-disrupting compounds such as pesticides (e.g., dichloro-diphenyl-trichloroethane; DDT) and plasticizers (e.g., bisphenol A; BPA), phytoestrogens are generally viewed as natural compounds that exert health benefits. In fact, soy isoflavones have been proposed as natural selective estrogen receptor modulators (SERM), which may be useful in the treatment or prevention of cardiometabolic diseases and estrogen-sensitive cancers. Soy isoflavones are present in foods in less bioavailable forms. After ingestion by humans, the gut microbiome can modify the bioavailability of soy isoflavones by metabolizing them into more biologically active forms [15,16]. An individual’s ability to produce (*S*)-equol, one of the soy isoflavone metabolites of daidzein, has been hypothesized to be critical for obtaining the health benefits from a soy-rich diet due to its high estrogenic potency [17]. This hypothesis supports the important role of the gut microbiome in isoflavone metabolism. However, only ~30–50% of humans are capable of converting daidzein to equol. Interestingly, it has been estimated that as many as 55% of individuals in Asian populations are equol-producers, compared to only 20–35% of individuals in Western populations [18]. This discrepancy is likely due to the large interpersonal variations in the gut microbiome and differences in habitual diets among different populations.

In this review, we summarize the current knowledge of the microbial conversion of the soy isoflavone daidzein to equol, and how this highly estrogenic isoflavone metabolite is associated with cardiometabolic health in postmenopausal women. Lastly, because of the large interpersonal variation in equol-producing capacity and the issue that rodents are efficient equol-producers, limitations of previous research and possible solutions for proof of principal testing are discussed [19,20]. Due to the important role of the gut microbiome in equol production, modulation of the gut microbiome may be used as a potential approach to maximize the estrogenic potential of soy isoflavones and improve the health of postmenopausal women. 

## 2. Absorption and Metabolism of Soy Isoflavones

Soybeans are rich in isoflavones, with daidzein and genistein being two of the most commonly studied compounds exerting estrogenic activities. Soy isoflavones usually exist in food as biologically inactive β-D-glycoside forms, genistin and daidzin. Glycoside forms of soy isoflavone have low bioavailability and are poorly absorbable in the gastrointestinal tract due to their large hydrophilic structures [16,21,22]. These glycosides can be deconjugated by β-glucosidases produced by the intestinal bacteria to form the unconjugated isoflavones or aglycones, daidzein and genistein [15,23]. The aglycone forms of isoflavones can then be absorbed and further metabolized in the liver [24]. Liver enzymes such as UDP-glucuronosyltransferase and sulfotransferase can form glucuronidated and sulfated forms of these isoflavones, respectively, that are later excreted in the bile and passed into the intestinal lumen [24,25]. Daidzein can be metabolized in the lumen by intestinal bacteria into the metabolites equol and *O*-desmethyl-angolensin (*O*-DMA) [15,26]. Equol is a phytoestrogen that has the strongest binding affinity to ERβ among all soy isoflavone metabolites [14,27,28,29,30]. Unlike daidzein, equol has a chiral center and therefore exists in two distinct diastereo-isomers: (*R*)- and (*S*)-equol. (*S*)-equol is the form exclusively produced by intestinal bacteria, and exhibits a much higher binding affinity for ERβ compared to (*R*)-equol [31]. 

Not all individuals have the intestinal bacteria necessary to produce equol and/or *O*-DMA from daidzein. Large interpersonal variation in the gut microbiome has resulted in only 30–50% of the total population harboring equol-producing bacteria within their gut microbiome, whereas 80–90% of individuals are estimated to be *O*-DMA-producers [32]. Bacteria with equol-producing capacity are distinct from those that can produce *O*-DMA from daidzein [33,34]. Therefore, equol- and *O*-DMA-producing phenotypes are independent of each other, meaning that the ability to produce *O*-DMA does not prohibit the equol-producing capacity of an individual [35]. 

The significant interpersonal variation of equol production is thought to contribute to the inconsistent outcomes in clinical trials assessing the health benefits of soy consumption. Some variations in equol production and levels of equol observed in the serum of equol-producers can be attributed to differences in gut microbiome composition, diet, lifestyle, and possibly sexual dimorphism. Some published data even suggest that within an individual, the degree of equol production can change over time [36,37]. The use of antibiotics has been shown to negatively impact equol production when the abundance and activity of equol-producing bacteria are compromised [38,39]. The presence and activity of equol-producing bacteria in the gut microbiome can both influence the equol-producing capacity of an individual, with the activity being affected by substrate availability due to differences in dietary soy isoflavone intake and the abundance of other bacteria that can aid in the production of equol within a given ecosystem [40]. Other dietary factors, such as a high intake of dietary fat, can have a negative impact on the ability of intestinal bacteria to produce equol, leading to a lowered level of serum equol concentration in the host [41,42]. Levels of carbohydrates entering the large bowel can also potentially impact equol production. Using a dynamic in vitro model of the human colon, equol production has been shown to increase when a higher level of starch exists within the system [43]. Interestingly, previous studies have suggested that the habitual diet of equol-producers tends to have higher carbohydrate as percent energy inclusion than that of non-equol-producers [41]. Lifestyle choices such as smoking, which can affect the gut microbiota, have also been found to negatively affect equol production [44,45]. 

Sex differences in equol production are less clear. In rats, females produce a higher concentration of equol compared to males after oral administration with the same bolus of soy, and this sexual dimorphism is consistent among three different sources of soy isoflavones containing varying amounts of daidzein [46]. In a small human clinical trial, the proportion of equol-producers is similar in men and women (~30% of all subjects); however, women are more likely to become equol-producers after chronic soy consumption compared to men [47]. Other studies have shown no sex differences in equol production [48,49,50,51]. Many confounding factors exist in humans that may influence equol production, such as genetic, microbial, dietary and behavioral differences, and therefore contribute to the challenging assessment of sexual dimorphism in equol production. 

Currently, longitudinal studies tracking absorption of daidzein and production of equol before, during, and after menopause transition do not exist. Therefore, it is unclear whether changes in endogenous ovarian hormone production throughout menopause transition affect gut bacterial production of equol in humans. Rodent data suggest that ovariectomy does not impact equol-producing capacity [52]. However, aging and interpersonal variations of the gut microbiome are critical factors in the equol production of women during menopause transition that do not exist in cross-sectional studies performed in conventionally raised rodent models. Rigorous and long-term human trials are needed to elucidate the interactions between endogenous mammalian estrogen synthesis and microbial synthesis of the estrogenic compound equol. Critically, dietary factors need to be carefully documented or controlled, as estrogen fluctuations are known to affect satiety and eating behavior. The establishment of an animal model with fewer confounding variables may be beneficial for elucidating the impact of these biological determinants on equol production, including sex differences and changes in sex hormone status.

## 3. Bacterial Equol Production

Intestinal bacteria play a significant role in the metabolism of soy isoflavones and the production of equol, as humans do not possess the enzymes necessary to carry out this conversion. Some efforts have been made to identify equol-producing bacteria from the gut microbiota (Table 1). So far, findings suggest that most equol-producing bacteria belong to the family *Coriobacteriaceae*. Genera within the *Coriobacteriaceae* family that have been shown to have equol-producing capacity include *Adlercreutzia, Slackia*, *Eggerthella*, *Paraeggerthella, Asaccharobacter,* and *Enterorhabdus,* [53,54,55,56]. Some genera outside of the *Coriobacteriaceae* family have also been identified, including certain species and strains within genera *Lactobacillus, Lactococcus,* and *Bifidobacterium* [54,57,58,59,60,61]. 

**Figure 1:**
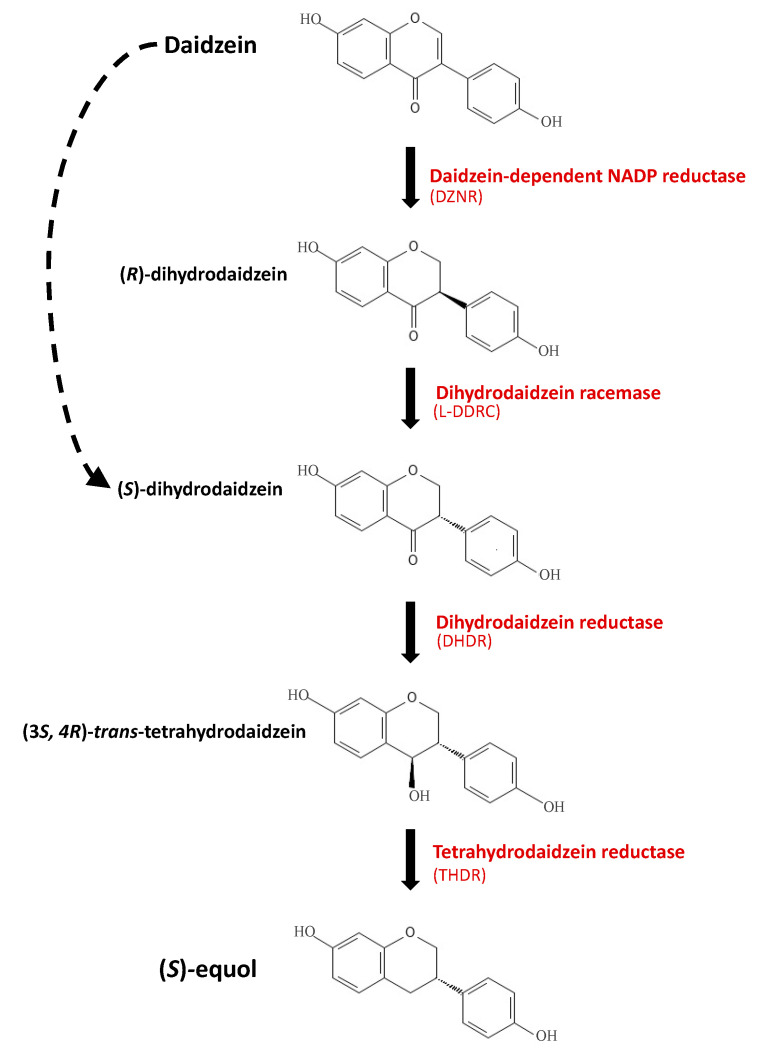
Diagram illustrating the current knowledge of the bacterial conversion of the soy isoflavone daidzein into the metabolite (*S*)-equol. Metabolite names are noted in black, and enzyme names and abbreviations in red. The dotted arrow indicates a possible alternative to the pathway, bypassing the use of the racemase enzyme.

The microbial conversion of daidzein to equol is a multi-step process that requires three essential reductase enzymes and can be aided by a racemase enzyme (Figure 1). These reductase enzymes responsible for the conversion of daidzein into equol were first discovered in the equol-producing strain *Lactococcus garvieae* 20–92, and have since been identified in other known equol-producing strains such as *Slackia isoflavoniconvertens*, *Eggerthella* sp. YY7918, and *Adlercreutzia equolifaciens* DSM 19450 [61,63,66,70,71,72]. The first step in this conversion uses a daidzein-dependent NADP reductase (DZNR), encoded by the *dzr* gene, which converts daidzein into (*R*)-dihydrodaidzein [61,63]. Early publications on the DZNR enzyme suggest that a racemic mixture of both the (*R*)- and (*S*)-form of dihydrodaidzein is produced during this process; however, more recent studies using a highly purified form of DZNR enzyme from *Eggerthella* sp. YY7918 suggest that only (*R*)-dihydrodaidzein is produced [61,63,66,73]. The second step in the conversion uses a dihydrodaidzein racemase (DDRC), which converts (*R*)-dihydrodaidzein into (*S*)-dihydrodaidzein [71,74]. The third step uses a dihydrodaidzein reductase (DHDR), encoded by the *ddr* gene, which converts (*S*)-dihydrodaidzein into (*3S*,*4R*)-*trans*-tetrahydro-daidzein [63,70]. This enzyme, DHDR, can also convert (*R*)-dihydrodaidzein into *cis*-tetrahydro-daidzein. The final step in the production of (*S*)-equol uses a tetrahydro-daidzein reductase (THDR) that has enantioselectivity for the (*3S,4R*)-*trans*-tetrahydro-daidzein [62,70,75]. The THDR enzyme is encoded by the *tdr* gene, and this enzyme completes the conversion of (*3S,4R*)-*trans*-tetrahydro-daidzein into (*S*)-equol [63,70]. These genes are inducible by the presence of daidzein in the environment [53,63].

There are still some uncertainties when it comes to the biotransformation of daidzein into equol. Among the four enzymes involved in the synthesis of equol, the racemase enzyme DDRC is the least studied, and its importance during this biotransformation is still unclear [76]. Reports have shown that when only the three reductase genes are cloned into a non-equol producing strain of bacteria, production of equol is still observed, but at much lower levels than when the racemase gene is cloned in combination with the other three reductase genes [61,71,77]. This suggests that the racemase enzyme aids in the production of equol, but it is not essential since equol can be produced at low levels in the absence of this racemase enzyme. Due to the enantioselectivity of the THDR enzyme and the exclusive production of (*R*)-form of dihydrodaidzein from daidzein, it is unclear how the production of equol occurs without the presence of this racemase enzyme. It is possible that some equol-producing strains can produce a racemic mixture of dihydrodaidzein from daidzein, as suggested in earlier publications [61,63,66,73]. The presence of this racemase enzyme and its ability to increase the efficiency of equol production may partially explain the interpersonal variation of equol production observed in humans.

Bacterial strains that possess all three equol converting reductase genes and the racemase gene, such as *Lactococcus garvieae* 20–92, *Slackia isoflavoniconvertens,* and *Adlercreutzia equolifaciens,* can carry out the full conversion of daidzein into equol on their own in the presence of daidzein [53,61,63]. Some bacterial strains, however, lack the ability to fully convert daidzein to equol due to the lack of one or two of these key enzymes [53,57,62,64,65,67,68]. Therefore, multiple strains may be needed within an ecosystem to collaboratively convert daidzein into equol [67]. Notably, the presence of equol converting genes is strain dependent. Even within a known equol-producing species, there can be unique strains that lack these genes and cannot produce equol [69]. The varying presence of these genes highlights the importance of the gut microbiome in modulating the bioavailability of soy, due to the dependency on these exclusively microbial enzymes with equol-producing capacity. The species of equol producing strains present within a gut microbiome, as well as the abundance of these strains, can impact equol production. Furthermore, other members present within the microbial community can facilitate or hinder equol production; therefore, the identification of useful microbial signatures of each unique human gut ecosystem will be useful when attempting to maximize the estrogenic potential of soy isoflavone daidzein for personalized nutrition.

## 4. Soy Isoflavones & Equol in Postmenopausal Women: Clinical Implications

### 4.1. Obesity and Diabetes

Obesity is one of the most prevalent diseases that leads to multiple metabolic dysfunctions such as insulin resistance, T2D, cardiovascular diseases, and cancer. Globally, 37% of men and 38% of women were obese in 2013, which was about 10% higher than in 1980 [78]. The increase in the prevalence of obesity and overweight in adults was higher than in children, and higher in women than in men [79]. Being overweight and obese was estimated to cause 3.4 million deaths in 2010 worldwide [80]. Total healthcare costs related to obesity are predicted to double every decade and are estimated to be about $900 billion by 2030 in the U.S [79].

A higher prevalence of obesity was observed in women of postmenopausal age than those of premenopausal age in 2009–2010 [81]. Regardless of race and ethnicity, 66–74% of the women at postmenopausal age are overweight or obese in the U.S., compared to a prevalence of 56% at premenopausal age [81]. During the menopause transition, women gain 2–2.5 kg of body weight and experience unfavorable fat redistribution, leading to the occurrence of central obesity [82]. Postmenopausal women are five times more likely to have central adiposity than premenopausal women, and abdominal obesity is highly prevalent in postmenopausal women. This alteration in fat deposition is likely to be due to the loss of the ovarian hormone estrogen. Excess visceral fat developed in obesity has been shown to secrete more adipokines and chemokines that promote insulin resistance, macrophage infiltration and activation, and is associated with elevated inflammatory markers such as interleukin 6 (IL-6) and tumor necrosis factor-α (TNF-α) [83]. A meta-analysis of >100 randomized trials in postmenopausal women concluded that the use of estrogen supplementation, with or without progesterone, increases lean body mass and reduces abdominal fat, improves insulin resistance, and decreases blood pressure, suggesting that it may minimize the obese phenotype of this population, supporting the theory that estrogen deficiency contributes to obesity in postmenopausal women [84]. However, controversy exists regarding the cause of midlife weight gain in women, with some studies unable to find an association between menopausal status and fat mass in women [85,86,87]. 

Soy isoflavones, similar to estrogen, are capable of suppressing the transcription of lipoprotein lipase, reducing fat accumulation, and reducing the ectopic accumulation of triglycerides [88]. Supplementation of the soy isoflavone glycoside daidzin in mice fed a high-fat diet has been shown to inhibit weight and visceral fat mass gain through modulating leptin-mediated appetite regulation to affect fat accumulation [89]. Interestingly, the association between equol and leptin may be sex dependent. Among prediabetic and diabetic human subjects, lower circulating leptin levels and leptin/BMI ratios were found in equol-producing females, but not in males [90]. Inhibition of glycolysis and activation of lipolysis by daidzein was also shown in a rat adipocyte cell line where the conversion of glucose to lipids was inhibited in a dose-dependent manner [91]. This inhibition of lipid formation from glucose and enhancement of lipolysis in the adipocyte cells when supplemented with daidzein, demonstrates the role that daidzein plays in regulating glucose and lipid metabolism [91]. 

A major upstream regulator of glucose and lipid metabolism is the peroxisome proliferator-activated receptor γ (PPARγ), a ligand-dependent transcription factor [92]. Soy isoflavones have been shown to activate the PPARγ signaling pathway, therefore modulating inflammatory responses, adipogenesis, lipogenesis, and lipolysis [93,94,95,96]. The activation of PPARγ is critical for insulin sensitivity and glucose homeostasis [97,98]. A high-fat, high-sucrose diet supplemented with daidzein has been shown to activate PPARγ in mice, with lower adipose inflammation and improved insulin sensitivity compared to the same diet without daidzein supplementation [99]. A similar observation has been found in rats fed diets of varying soy isoflavone content, with a diet high in soy isoflavones able to improve lipid metabolism and glucose tolerance in both male and female rats [100]. Daidzein and equol can increase adipocyte differentiation and upregulate PPARγ in in vitro cell lines [98]. Furthermore, the same study demonstrated that daidzein increased insulin sensitivity by increasing insulin-stimulated glucose uptake through insulin-responsive glucose transporter 4 (GLUT4) [98]. Similar beneficial effects of soy isoflavones have been shown in controlled clinical trials where postmenopausal women with T2D were given soy proteins containing aglycone forms of soy isoflavones (132 mg/day of a mixture of 53% genistein, 37% daidzein, and 10% glycitein) [101]. After 12 weeks of intervention, soy isoflavones significantly decreased circulating fasting insulin levels, improved insulin resistance, and maintained long-term glucose stability. 

Pancreatic β-cells synthesize, store, and release insulin and anti-hyperglycemic hormones. In the early stages of T2D, β-cells begin to lose their functional mass, and the increased abundance of apoptotic β-cells ultimately leads to insulin resistance [102]. Soy isoflavones and equol can enhance the functionality of β-cells and exert anti-diabetic effects. In vivo analysis suggested that equol enhanced β-cell proliferation by 27% in mice compared to controls, and ex vivo analysis suggested that equol supplementation increased insulin secretion by 41% whereas daidzein supplementation did not significantly increase insulin secretion [103]. This amelioration of β-cell formation and function from equol was contributed through reducing cell death by suppressing alloxan-induced oxidative stress in INS-1 pancreatic β-cells [104]. Interestingly, (*R*)-equol did not demonstrate this protective effect [104]. In Zucker diabetic rats, (*S*)-equol supplementation has been shown to reduce carbohydrate response element-binding protein and thioredoxin-interacting protein signaling, delaying the onset of hyperglycemia and the failure of insulin secretion [105].

In humans, the anti-diabetic effect of soy has been suggested in epidemiological studies, although the link to equol remains unclear, as insufficiently consistent results were found in humans [106,107,108]. This lack of consistent reporting was likely to be due to the presence of other functional ingredients in the diet, interpersonal variations of the gut microbiome, different intestinal microbial responses to varying diets, as well as other genetic and environmental factors. Additionally, not all published clinical trials with soy intervention examine equol levels. An inverse association between urinary equol and the risk of T2D in adults was reported in China [109]. However, a significant association between the level of urinary phytoestrogen metabolites and T2D was not found in the Singapore Chinese Health Study [110]. In another study involving a multi-ethnic cohort, soy consumption was not shown to have any protective effect against the risk of T2D [111]. Discrepancies in these findings may be due to inconsistent measurement of equol concentrations, use of different types of biological samples, and different study designs. Thus, conclusions drawn from these studies should be evaluated with caution for accurate interpretation.

### 4.2. Cardiovascular Diseases

CVD is the leading cause of death in the United States (US) and the statement released by the American Heart Association in 2020 emphasized the significance of the menopause transition on the greater risk of CVD in women [112,113]. Epidemiological studies have suggested cardioprotective properties of soy, with soy intake being found inversely associated with cardiovascular-related death [114,115,116]. Similarly, preclinical studies have found that soy isoflavone supplementation significantly decreases atherosclerosis in monkeys and rabbits [117,118]. Soy supplementation can also decrease myocardial infarct size in ovariectomized rats, a model of human menopause [119]. However, the effects of soy supplementation on cardiovascular health assessed in prospective human studies are less conclusive. For example, a randomized control trial found that daily intake of 25 g of soy containing 91 mg aglycon isoflavone equivalents did not significantly reduce subclinical atherosclerosis progression in postmenopausal women [120]. Interestingly, maternal exposure to a soy diet reduces atherosclerotic lesions of the offspring by promoting anti-inflammatory responses, suggesting a transgenerational impact of dietary soy intake and adding another layer of complexity when assessing soy and cardiovascular health [121]. Soy isoflavones may lower the risk of CVD through several mechanisms: (i) altering systemic lipid and apolipoprotein profiles, particularly lowering cholesterol (ii) reducing inflammation, (iii) reducing oxidative stress, (iv) decreasing low-density lipoprotein (LDL) oxidation that contributes to atherogenesis, and (v) inducing nitric oxide (NO) production to prevent endothelial dysfunction. 

The cholesterol-lowering effect of soy was thought to be well documented, although ongoing debates exist with the US Food and Drug Administration (FDA)’s recent re-evaluation and proposal to revoke its health claim for soy protein and risk of coronary heart disease (see discussion by Petersen) [122]. Multiple meta-analyses have demonstrated the ability of soy to reduce serum total and LDL cholesterol, but with no impact on altering high-density lipoprotein (HDL)-cholesterol and triglycerides [123,124,125]. When considering the equol-producing status of the participants as a factor, a study assessing published data from three randomized clinical trials done in men and postmenopausal women showed that soy consumption leads to lower circulating LDL-cholesterol levels in both equol-producers and non-equol-producers [126]. However, data from this study suggest that equol-producers may benefit more from soy foods by maintaining higher levels of HDL-cholesterol than non-equol-producers. The high estrogenic potential of equol and its selective estrogen receptor modulating tendency has been speculated to be the mechanism of its ability to alter the circulating lipid profile. A recent systematic review and meta-analysis attempting to isolate the impact of various fractions of soy foods on serum lipids. Their data suggested that in postmenopausal women, soy isoflavones decreased serum total cholesterol and ApoB levels, whereas soy protein containing isoflavones not only reduced serum total cholesterol and ApoB levels but also total triglycerides and LDL-cholesterol [127]. This suggests that ingestion of whole foods may exert more benefits than isolated soy isoflavones alone, which could be due to differential bioavailability of isoflavones in various food matrices and/or the presence of other components in soy, such as oligosaccharides that can promote bacterial fermentation in the colon. Soy also contains lecithin, saponins, and dietary fiber that may benefit cardiovascular health through mechanisms independent of isoflavones. 

In addition to the traditional risk factors of CVD, such as hypercholesterolemia discussed above, inflammation exemplified by elevated C-reactive protein (CRP) is thought to be a non-traditional risk factor of CVD. Inflammation plays a prominent role in the development of atherosclerosis, which is the major cause of CVD. In postmenopausal European women, consumption of cereal bars enriched with genistein and daidzein (2:1 ratio, 50 mg of isoflavones/day) for 8 weeks coincided with a decrease in the circulating concentration of a pro-inflammatory marker CRP [128]. However, this dietary intervention did not improve other pro-inflammatory related biomarkers assessed, including monocyte chemoattractant protein 1 (MCP-1) and vascular cell adhesion molecule 1 (VCAM-1). A Dietary Approaches to Stop Hypertension (DASH) diet containing 30 g of roasted soy nuts led to an 8.9% decrease in circulating CRP and a 9.2% decrease in pro-inflammatory marker IL-18 than the control red-meat-containing DASH diet after 8 weeks of dietary intervention in postmenopausal women with metabolic syndrome [129]. This study also observed a markedly greater level of NO associated with soy nuts compared to the red meat diet. Daily consumption of 80 mg of soy isoflavone (55% genistein, 23% daidzein, and 14% glycitein) for 24 weeks reduced circulating CRP, IL-6, and TNF-α in patients with ischemic cardiomyopathy, a major cause of heart failure [130]. The anti-inflammatory response observed in this study is thought to be mediated through an erythroid-derived 2-like 2 (Nrf2)-activating mechanism, which mediated a superoxide dismutase (SOD) antioxidant response. Seven weeks of dietary daidzein supplementation can increase antioxidant SOD activity and decrease the content of oxidative stress marker malondialdehyde in streptozotocin (STZ)-induced diabetic rats [131]. 

Other than their anti-inflammatory and anti-oxidative properties, isoflavones have also been shown to inhibit LDL oxidation and protect the endothelial cells against the cytotoxic effects of oxidized LDL [132,133]. Oxidative damage to LDL is thought to be the hallmark in the pathogenesis of atherosclerotic vascular disease. In a human feeding trial, consuming soy bars that contain 21 mg of daidzein and 36 mg of genistein daily for 2 weeks significantly reduced oxidative susceptibility of LDL via an in vitro copper-ion-induced LDL oxidation test [134]. Similarly, daily feeding of soy burger patties that contained 21.2 mg of daidzein and 34.8 mg of genistein for 17 days reduced oxidative susceptibility of LDL compared to human subjects who consumed a lower amount of soy isoflavones (0.9 mg of daidzein and 1.0 mg of genistein) [135]. 

NO, synthesized in the vascular endothelium, is a vasodilator that modulates vascular tone and blood pressure. Dysfunction of the NO pathway contributes to the pathophysiology of CVD. Daily consumption of 30 g of roasted black soybeans (48.4 mg genistein, 78.1 mg daidzin, 0.5 mg daidzein, 0.5 mg glycitin, and 0.1 mg glycitein) for 8 weeks significantly improved vascular stiffness by increasing NO production and decreased markers of oxidative stress in healthy women [136]. Soy isoflavone daidzein has also been shown to improve vascular function through the NO pathway in male rats [137,138]. In a macrophage cell line activated with interferon γ plus lipopolysaccharide, both genistein and daidzein attenuated NO production and inhibited the secretion of proinflammatory marker TNF-α in a dose-dependent manner [139]. Similarly, in lipopolysaccharide-stimulated astrocytes, equol has been shown to attenuate NO production through the mediation of the G protein-coupled receptor 30, a non-genomic estrogen receptor, pathway [140]. 

## 5. Current Limitations and Future Research of Soy Isoflavones and Equol: Developing Necessary Model Systems for Maximizing Estrogenic Potential of Daidzein

Soy consumption has the potential to provide health benefits, likely more so in those whose intestinal bacteria are capable of producing equol from the soy isoflavone daidzein. Human clinical trials have inherent limitations that are difficult to control experimentally, such as sex and sex hormone status, genetics, lifestyle, and diet. All of these factors can hinder our ability to accurately validate how essential an individual’s equol-producing capability is in soy-related health-promoting attributes. Moreover, the gut microbiome of an individual is as unique as a fingerprint, which makes it challenging to first identify the importance of this particular intestinal bacterial-dependent conversion, as well as the involvement of governing microbial communities in human populations [141]. Rodents are efficient equol-producers, except those that are raised germ-free [142]. Therefore, soy feeding studies using conventionally-raised rodent models may only be relevant to a small and skewed population of humans. Developing a proper negative control (i.e., a mouse model with a gut microbiome that cannot produce equol) is critical to discern the benefits of consuming soy while being an equol-producer.

Germ-free rodents do not produce equol due to the absence of intestinal bacteria required to metabolize daidzein into equol [142]. However, living in a sterile environment leads to various developmental and physiological abnormalities, which makes germ-free rodents a poor control to use as non-equol-producers when examining physiological and metabolic outcomes. Gnotobiotic mice (i.e., germ-free mice colonized with a known microbial community), on the other hand, can provide a proper negative control as non-equol-producers, as long as their gut microbiome does not consist of microbial members with equol-producing capacity. Various approaches can be used in this effort to create non-equol producing rodents, e.g., colonizing germ-free mice with complex communities from human donors. This approach has been successfully demonstrated almost two decades ago by Bowey et al., whereby a gnotobiotic rat without any equol-producing capacity was created by colonization with a human microbiome of a poor equol-producer [143]. This approach using the human microbiome to inoculate germ-free mice has the advantage of being relevant to humans; however, confounding factors associated with the human gut microbiome exist. The unique signatures of the gut microbiome of an individual make comparisons and mechanistic investigation among communities difficult [141]. Further, the ecological dynamics of a complex community when encountering a novel equol-producing species may be individualized, which can make the discovery of next-generation probiotics with equol-producing capacity challenging. 

An alternative approach is to create synthetic bacterial communities by combining cultivated bacteria strains that mimic the human microbiome. Various synthetic bacterial communities which have been proposed to be suitable for this purpose are the Altered Schaedler Flora, Oligo-Mouse-Microbiota, Simplified Human Microbiota, among others [144,145,146,147,148]. This controlled approach allows customization when designing a microbiome with desired traits. Some benefits of using a synthetic community to colonize germ-free mice are community stability and reproducibility. Community stability is achievable for long periods of time using these communities, and the reproducibility of community colonization across animal facilities has been demonstrated when proper handling is employed to avoid contamination [147,149,150]. Synthetic communities with disparate equol-producing capabilities can be assembled and employed for proof-of-principal testing with minimal confounding variables influenced by the host. However, synthetic bacterial communities are generally much less complex than that of the human microbiome and often ignore other domains of life that inhabit the human gut, such as archaea, fungi, protists, and viruses. These members, although they exist less abundantly, can play significant roles within the ecosystem in impacting the physiological outcomes of the host. Therefore, caution must be taken when interpreting results from these studies utilizing simple synthetic communities.

Exogenous production of equol from chemical synthesis or in vitro bacterial fermentation used as a dietary supplement has gained some attention. A high level of oral equol supplementation appears to have some success in reducing cardiovascular risks and bone resorption for non-equol-producers [59,151,152]. However, the rapid absorption and pharmacokinetics of oral equol supplementation are likely to differ significantly from endogenous production within the gastrointestinal tract [153,154]. In rats, oral equol supplementation leads to a markedly higher level of the glucuronidated form of equol in serum within a much shorter period of time compared to daidzein supplementation [154]. These data suggest that equol supplementation, although it does not rely on the gut microbiome and may potentially benefit non-equol-producers, differs in equol metabolism compared to equol generated through endogenous microbial synthesis and may exert differential health benefits. Furthermore, a recent study reported that equol supplementation provides no cardiovascular benefits in non-equol producing men, unlike those shown in non-equol producing women [155]. The development of an appropriate model system is essential to discern these conflicting results and critically assess the differences between endogenous synthesis vs. exogenous production of equol. Moreover, developing novel strategies to maximize the nutritional efficacy of soy foods by introducing equol-producing bacteria as next-generation probiotics may significantly promote the health of equol non-producing individuals.

## 6. Conclusions

The soy isoflavone daidzein may provide cardiometabolic benefits to postmenopausal women, especially when metabolized to equol by an individual’s intestinal bacteria. However, inconsistent discoveries in human clinical trials warrant a more controlled model for mechanistic studies in vivo. Due to the nature of the human gut microbiome, significant interpersonal variations can lead to conflicting outcomes, making it difficult to interpret the benefits associated with the microbial metabolism of soy isoflavones on top of other inherent confounding factors present in clinical trials. Moreover, the highly efficient equol-producing capacity of natural rodent microbiomes makes the relevance of soy feeding studies using conventionally raised rodent models likely limited to only a subpopulation of humans. Model systems with proper negative controls that provide higher accuracy are needed to mechanistically identify the necessity of equol production in human health and understand microbe–microbe interactions between a governing human microbial community and equol-producing bacterial strains. Successful establishment of these model systems can pave the path to maximize equol production through modulation of the gut microbiome, and provide potential therapeutic strategies to improve the cardiometabolic health of postmenopausal women.

## Figures and Tables

**Table 1:** A summary of the bacterial strains referenced and discussed in this review. The second column indicates specific steps of the daidzein to equol conversion that each strain is capable of carrying out. This is not an inclusive list of all known daidzein converting and/or equol producing bacterial strains.

Bacterial Strain	Conversion	Source
*Adlercreutzia equolifaciens strain* DSM 19450	Daidzein → Equol	[53]
*Lactobacillus intestinalis*	Daidzein → Equol	[54]
*Coriobacteriaceae* strain Mt1B8	Daidzein → Equol	[55]
*Eggerthella* sp. strain Julong 732	Dihydrodaidzein → Equol	[57,62]
*Bifidobacterium breve* strain 15700	Daidzein → Equol	[58]
*Bifidobacterium longum* strain BB536	Daidzein → Equol	[58]
*Lactococcus garvieae* strain 20–92	Daidzein → Equol	[60]
*Slackia Isoflavoniconvertens* strain DSM 22006	Daidzein → Equol	[63]
*Clostridium* sp. strain HGH6	Daidzein → Dihydrodaidzein	[64]
*Coprobacillus* strain TM-40	Daidzein → Dihydrodaidzein	[65]
*Lactobacillus* sp. Niu-O16	Daidzein → Dihydrodaidzein	[66,67]
*Eggerthella* sp. YY7918	Daidzein → Equol	[68]
*Adlercreutzia equolifaciens* strain W18.34a	No conversion of daidzein	[69]

## Data Availability

Not applicable.

## Abbreviations

BPA	bisphenol A
CRP	C-reactive protein
CVD	cardiovascular disease
DZNR	daidzein-dependent NADP reductase
DDT	dichloro-diphenyl-trichloroethane
DDRC	dihydrodaidzein racemase
DHDR	dihydrodaidzein reductase
ERβ	estrogen receptor beta
GLUT4	glucose transporter 4
IL-6	interleukin
LDL	low-density lipoprotein
MCP-1	monocyte chemoattractant protein 1
*O*-DMA	*O*-desmethylangolensin
PPARγ	proliferator-activated receptor γ
SERM	selective estrogen receptor modulators
THDR	tetrahydrodaidzein reductase
TNF-α	tumor necrosis factor-α
T2D	Type 2 diabetes
VCAM-1	vascular cell adhesion molecule 1
